# Variation of Seasonal Temperature and Water Stress Modify the Composition of Leaf Essential Oils in Grapefruit (*Citrus* x *aurantium* var. *paradisi*), Sweet Orange (*Citrus x aurantium* var. *sinensis*) and Clementine (*Citrus x aurantium* var. *clementina*) Trees

**DOI:** 10.1002/cbdv.202503648

**Published:** 2026-04-21

**Authors:** François Luro, Mathieu Bernardini, Margie Rovinalti, Félix Tomi

**Affiliations:** ^1^ UMR AGAP Institut Univ Montpellier, CIRAD INRAE Institut Agro San Giuliano France; ^2^ Rémy Cointreau – Les Molières Saint‐Barthélemy‐d'Anjou France; ^3^ UMR SPE 6134 – Université de Corse – CNRS Ressources Naturelles Ajaccio France

**Keywords:** environment, (E)‐2‐hexenal, leaf maturity, limonene, linalool

## Abstract

Mainly grown for food, citrus trees are also highly used as sources of aromatic essential oils (EOs) by the cosmetics and perfume industries. The EOs composition is highly complex with more than hundred compounds. It varies according to cultivation techniques, climatic conditions, and environmental stresses, whether of biotic or abiotic origin. The current study analyzed the effect of climatic variation, as well as water stress and leaf age, on the composition of leaf EO (LEO) in three citrus varieties (sweet orange, grapefruit, and clementine). From September to March, sabinene levels rose continuously across all three citrus varieties. Water stress induced a strong momentary increase of limonene. Annual variations in LEO were less significant, and particularly concerned limonene, sabinene, terpinen‐4‐ol and linalool. Whatever the citrus fruit, leaf maturity status was found to be an important factor in the quantitative and qualitative variation of LEO composition. The quantity of (E)‐2‐hexenal increased during water deficit and colder months of winter. These results demonstrate that environmental conditions have an impact on the composition of LEO and most probably, on its aromatic profile. The role of limonene in citrus management of water stress and other environmental factors needs more investigation.

## Graphical abstract

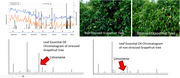

## 1 Introduction

Citrus can adapt to a wide variety of environments and climates, with cultivation covering more than 10 million hectares, mainly in areas between the two 40th parallels. The choice of varieties and rootstocks, as well as cultivation methods, are crucial to the success of these adaptations. Climate affects citrus fruit quality, notably yield, sugar content, acidity, succulence, and color [1]. In addition to its use as a foodstuff, citrus production also supplies the food industry, cosmetics, therapy, and perfumery, through the extraction of essential oils from zest, flowers (neroli) and leaves (petitgrain). Among essential oils, orange and lemon zest oils (PEO) are the most abundant, with 49,000 and 9,000 T respectively (2019, FranceAgriMer sources, https://www.franceagrimer.fr), as they are by‐products of the juice industry. Neroli (around 4 T) and petitgrain (around 400 T) are most often produced from sour oranges (*Citrus x aurantium* var*. aurantium*) and their use is limited to perfumery and aromatherapy. The biological role of essential oils in the plant is not very well known, although the synthesis of certain compounds seems to be induced by the presence of a pathogen or abiotic stress. It has been proposed that these compounds may act as chemical messengers or thermal regulators, and are also attractive to pollinating insects.

The composition of citrus leaf and zest essential oils varies according to environment or geographical origin [2]. The yield and composition of PEO varies during fruit development and the different stages of ripening, with very marked instability during the first two phases [3]. Few studies trace a direct relationship between variation in essential oil composition and climate. Growing conditions, varieties and physiological stages of the fruit are difficult to compare from one country to another. Leaves would be more appropriate for this study, since unlike fruit, they develop rapidly (within a few weeks) and are perennial. The composition of *Citrus aurantium* LEOs changes throughout the year [4]. In this study, LEO composition was analyzed at 5 dates of the year (January, April, July, September and November). The greatest variation observed was in linalyl acetate, which rose from 0.77% to 24.77% between April and November. The summer LEO profile had the highest number of compounds, suggesting to the authors a protective role against environmental threats. It is regrettable, however, that this study did not consider tree phenology as a potential factor of variation, such as leaf age, flowering, fruit load, and development.

The period from April to September is particularly energy‐intensive for citrus. Flowering, which generally takes place in April in the Mediterranean basin, follows a vegetative shoot that began a month earlier. The number of flowers on a tree can reach several hundred thousand. After fertilization, the fruit enters phase I of its development, corresponding to cellular multiplication, which lasts around 2 months and ends with a significant drop in small fruit (early abscission), helping to balance the tree's fruit production. Phase II corresponds to cell enlargement and begins with the entry of water into the fruit cells and the synthesis of soluble sugars (sucrose, fructose, glucose), and organic acids (mainly citric acid). Fruit size increases most during the first two months of Phase II (July and August). By mid‐September, the fruit is considered to have reached over 80% of its ultimate size. This marks the beginning of phase III, the ripening phase. It is at this point (late September—early October) that the composition of orange peel essential oils (PEO) stabilizes [3]. This stabilization may only be apparent, as limonene is dominant (over 92% in orange, sour orange, and clementine). It is therefore very difficult to measure variations in PEO profiles potentially linked to climate. In this respect, LEOs are more appropriate for measuring seasonal climatic effects, as limonene is not dominant.

Many factors can modify the EO composition, such as growing location, cultivars (genetic variability), ripening stages, storage conditions, and extraction methods [5]. Biotic and abiotic stress are also factors that induce the synthesis of new volatile compounds or modify the EO composition [6]. The terpenoids are family of volatile organic compounds (VOC) abundantly emitted by stressed plants [7, 8]. Water deficit stimulates biosynthesis of (E)‐2‐hexenal, an aliphatic aldehyde synthesized after membrane denaturation, in stressed leaves [9]. Some authors suggested that (E)‐2‐hexenal can act as chemical endogenous signal inducing abiotic‐responsive genes [10]. Other studies profiled citrus VOCs in response to winter flooding and salinity [11]. Flooding transiently caused large and rapid emissions of limonene in the leaves and in young and mature fruits.

The aim of our study is to analyze the LEO composition according to the climatic variation over the season (between August and April), between years and also in relation to a water stress. During the September‐Marsh period, the fruit was left on the trees and no vegetative development flow was observed (no production of new leaves). The cessation of sampling was conditioned by the appearance of new vegetative shoots in the month following the last sampling (March) and the emergence of flower buds in April. The LEO composition between adult and young leaves has been also assessed. Various climatic parameters were scored by a weather station located near the orchard at the INRAE research station in San Giuliano (12) during the sampling dates.

## 2 Results

### 2.1 Climatic Variations and Tree Condition During the Sampling Period

In September 2023 leaf harvest was carried out on trees showing marked symptoms of water shortage, that is leaf curling and epinasty. Despite the September rains, albeit in small quantities, the thirst symptoms persisted until the second sampling on October 13, 2023. The trees returned to normal appearance by the end of October.

Given that irrigation was stopped 1 month before the second sampling (September 2023), the only water supply was based on rainfall. Cumulative rainfall in the month preceding the first sampling was very low (7.5 mm), then increased progressively between 2 samplings, reaching 137 mm by the seventh sampling, between February and March 2024 (Table 1). Meanwhile, average temperatures remained high over the period from mid‐August to mid‐October (>20.0°C), then gradually decreased to around 10.0°C between December and March. PAR (Photosynthetically active radiation and PET (Potential evapotranspiration) were highest in August‐September and lowest in December‐January.

**TABLE 1:** Corsican climatic parameters during a period of 30 days before leaf sampling and LEO composition analysis.

	2022	2023	2023	2023	2023	23‐24	2024	2024	2024
	Aug‐Sep	Aug‐Sep	Sep‐Oct	Oct‐Nov	Nov‐Dec	Dec‐Jan	Jan‐Feb	Feb‐Mar	Aug‐Sep
Precipitations *(sum in mm)*	41	7.5	27.0	24.0	68.5	54.5	10.0	137.0	27.0
Temperature *(mean in°C)*	24.1	24.0	20.5	17.7	12.3	10.2	9.6	10.7	25.0
PAR^1^ *(mean in µmol.m‐^a^.s‐1)*	1239	1069	730	411	330	271	397	504	885
PET^a^ *(mean in mm)*	4.2	4.7	2.0	0.8	0.4	0.7	1.3	2.4	4.3

^1^ Photosynthetically active radiation.

^a^ Potential evapotranspiration.

In 2022 and 2024, irrigation was maintained all summer and until the end of September, so rainfall over the August‐September period is not comparable, although it is much higher in 2022 and 2024 than in 2023, (Table 1). The trail in 2024 was therefore in water deficit over this period. Temperatures, PAR and PET were equivalent to those of the August‐September 2024 period.

### 2.2 The Composition of LEO Over the Autumn‐Winter Period Following the Water Stress

**FIGURE 1:**
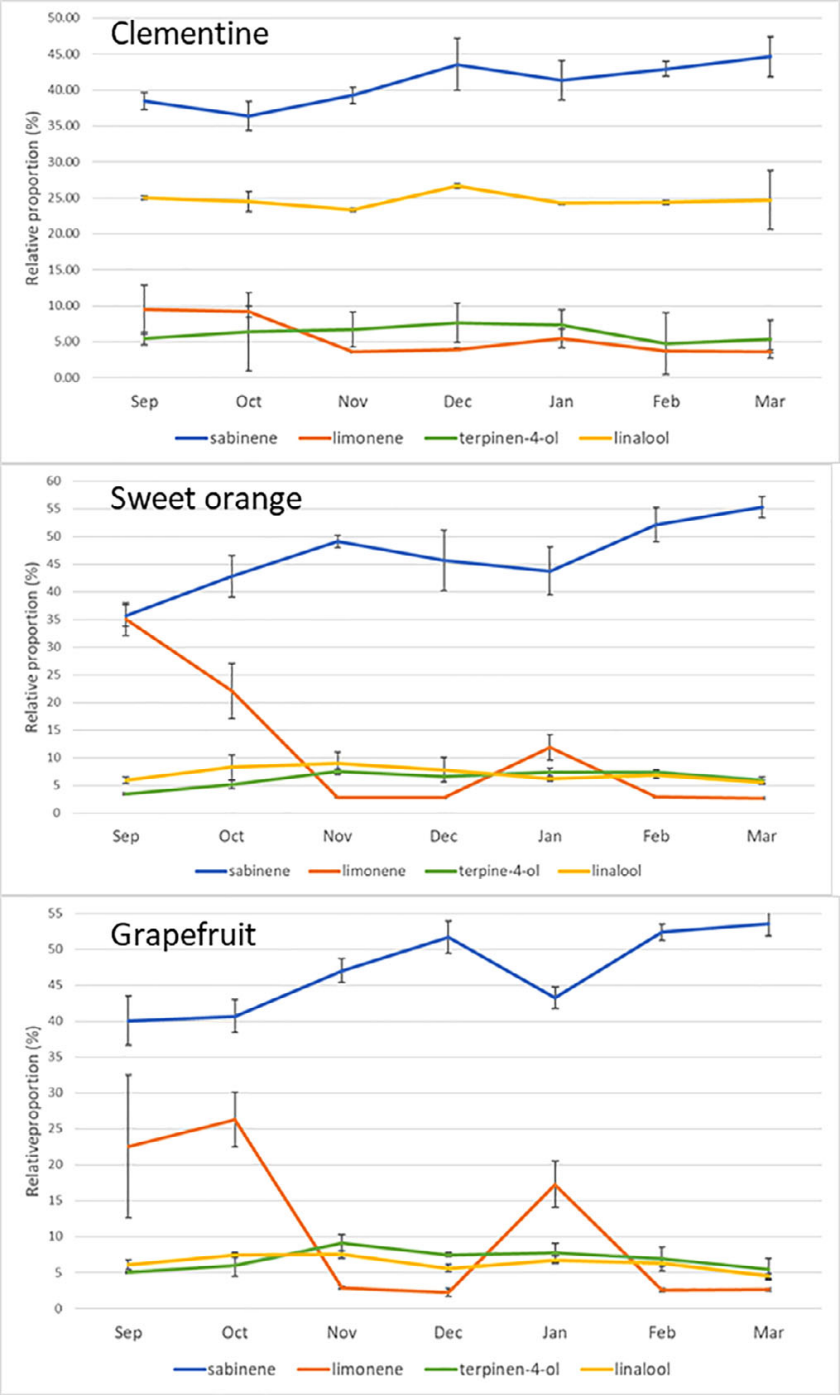
Variation of the relative proportion (%) of sabinene, limonene, linalool, and terpine‐4‐ol from September, 12 2023 to March 15, 2024 for clementine, sweet orange and grapefruit; the vertical bar represents the SD.

LEO composition varied over the sampling period from September 2023 to March 2024, particularly in orange and grapefruit (Figure 1 and Table S1). Through all leaf essential oil analyses, 52 unique compounds were found in oranges, 47 in grapefruit, and 49 in clementine's. Two major compounds, sabinene and limonene, fluctuated. Despite monthly variations, the proportion of sabinene increased over the period, starting at around 40% and ending at around 52% in March.

The limonene curve was more chaotic, with, for example, in grapefruit, a relatively high proportion in September and October (> 22%), followed by a clear decrease in November, stabilization in December (3%), a clear rise in January (up to 9%) and then a return to the same November and December values in February and March. Very similar kinetics were found in orange trees, with a very high limonene content in September (>35%). The increase in January was less marked than in grapefruit, but in both citrus fruits, the increase in limonene was accompanied by a decrease in sabinene. In clementine, the proportion of the 4 major compounds were more stable over the months, but nevertheless with an increase in sabinene from 38 to 45% between September and March, and a limonene level of 10% in September and October, which then decreases to around 5% for the rest of the season. While it does not exceed 10% in grapefruit and orange, linalool in clementine represented 25% of compounds throughout the observation period. A slight increase in terpinen‐4‐ol was observed between September and November, followed by a slight decrease from February onwards, whatever the variety.

**FIGURE 2:**
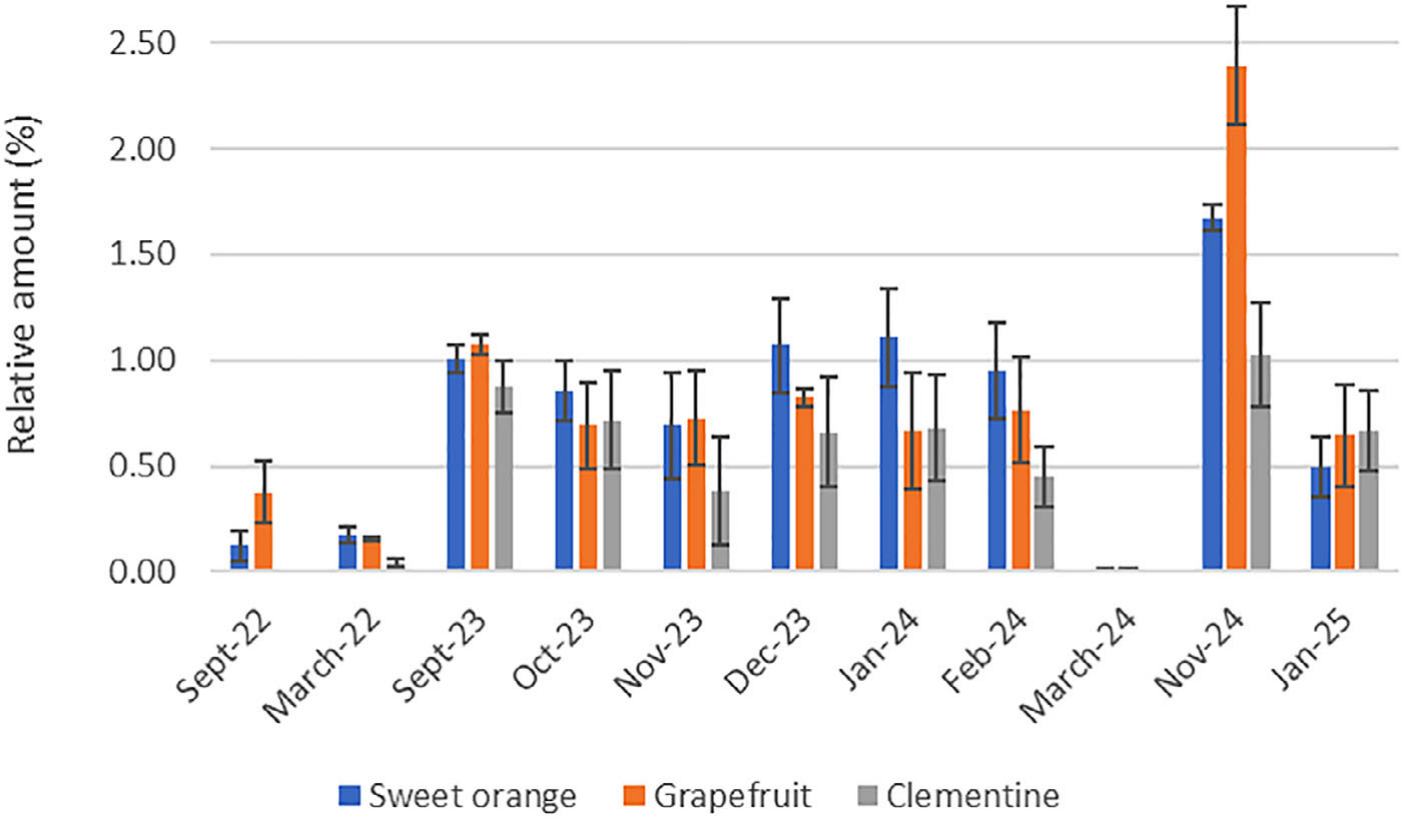
Proportion (%) of (E)‐2‐hexenal over months and years for clementine, grapefruit, and sweet orange. The vertical bars represent the SD.

In clementine LEO, few minor compounds varied over the period of August 2023‐March 2024 (Table S1). (E)‐2‐hexenal was only absent in March, and β‐elemene and γ‐elemene were only present in September. In orange leaf, the variation of (E)‐2‐hexenal was higher, with a presence of 0.50% to 1.67% during the autumn‐winter period, and a quantity of less than 0.17% in the other months (Figure 2). In grapefruit, with the exception of September 2022, the same trend was observed than in sweet orange, with a higher quantity of (E)‐2‐hexenal in the autumn‐winter months, and no more than 0.17% in March 2023 and March 2024. Its highest level was observed in November 2024 (2.39%). The increase of (E)‐2‐hexenal observed in autumn‐winter period was not related to the water stress because its level was similar in January 2024 and January 2025.

The proportion of p‐cymene varied over the months (0.48% in February, but no more than 0.05% in the other months). Similarly, in the grapefruit LEO, few minor compounds varied, and only citronellyl acetate showed a proportion of 0.56%, whereas it does not exceed 0.05% in all other months. Nerol was absent in September and October, and as with clementine, hexanal was absent in March. The number of compounds varying in the ranger LEO was greater than for the other two varieties. α‐phellandrene, δ‐3‐carene, terpinolene, citronellal, citronellol, and geranial were present in smaller quantities in September and October than in the other months. Nerol was only present in February and March, and γ‐cadinene only detected from September to November.

### 2.3 Variation of LEO Composition Between Years

The composition of LEO differed markedly between September 2022 and September 2023, particularly for the main compounds (> 1%) (Table 2 and Table S1). The proportion of four to five compounds were statistically different for each cultivar. Sabinene was higher in September 2022 for the three citrus and also linalool for grapefruit. Limonene and (E)‐2‐hexenal were in higher proportions in September 2024 when the trees were in water stress. There was little variation in minor compounds, although p‐cymene in clementine LEO was present in greater quantities in September 2022, and certain compounds were present in one year but not the other, such as α‐sinensal and octanal in grapefruit and nerol in sweet orange

**TABLE 2:** Mean values (± SD) of the proportion (%) of major LEOs compounds (>1%) from leaves harvested in September 2022 and September 2023 for the 3 citrus varieties: Each value derived from biological triplicates. Statistical significance between young and mature leaves was assessed using Student's *t*‐test (for normally distributed data) or Mann‐Whitney test (for non‐parametric data). Significant differences (*p* < 0.05 in light gray, *p* < 0.01 in dark gray) are highlighted in the table.

	Clementine		Grapefruit		Sweet orange	
Compounds	Sept‐22	Sept‐23	Sept‐22	Sept‐23	Sept‐22	Sept‐23
(E)‐2‐hexenal	0	0.88 ± 0.12	0.76 ± 0.15	1.11 ± 0.07	0.12 ± 0.07	1.00 ± 0.06
α‐pinene	1.28 ± 0.13	1.56 ± 0.01	1.83 ± 0.16	1.83 ± 0.22	2.01 ± 0.05	1.35 ± 0.09
(*E*)‐β‐ocimene	2.87 ± 0.50	3.65 ± 0.35	7.47 ± 0.25	7.82 ± 1.08	5.21 ± 0.19	3.04 ± 0.38
sabinene	46.15 ± 2.40	38.47 ± 1.21	50.46 ± 0.86	40.64 ± 2.65	54.55 ± 0.61	35.68 ± 1.95
δ‐3‐carene	2.38 ± 0.23	2.20 ± 0.60	0.03 ± 0.02	0.02 ± 0.00	2.68 ± 0.41	1.24 ± 0.23
limonene	3.83 ± 0.84	9.44 ± 3.43	3.16 ± 0.13	20.52 ± 7.82	1.91 ± 1.61	35.05 ± 2.96
linalool	25.13 ± 5.74	24.98 ± 0.84	10.69 ± 0.92	6.13 ± 0.09	7.15 ± 0.99	6.31 ± 0.78
terpinen‐4‐ol	4.88 ± 0.89	5.45 ± 0.23	3.5 ± 2.98	5.28 ± 1.04	4.68 ± 1.56	3.46 ± 0.15
β‐sinensal	0.65 ± 0.20	1.70 ± 0.45	1.30 ± 0.29	1.30 ± 0.16	1.38 ± 0.32	1.08 ± 0.38

The LEO compounds with relative proportions higher than 1% were compared between March 2023 and March 2024 (Table 3 and Table S1). Nine compounds showed significant and statistical differences. Five compounds were common between sweet orange and grapefruit with similar variations: the relative proportions of α‐terpinene and terpinen‐4‐ol were greater in 2024 than 2023 and those of β‐elemene, *(E)*‐β‐caryophyllene, and *(E)*‐phytol were greater in 2023. The proportion of the linalool in clementine was higher in 2024 than in 2023 (24.68% *vs*.14.49%), while the difference was inverted for grapefruit (4.48% *vs*. 8.43%). α‐terpinene and γ‐terpinene were in higher proportion in 2024 for grapefruit The quantity of α‐sinensal in clementine and β‐sinensal in sweet orange were higher in 2023. Among minor compounds, some were absent/present, such as δ‐cadinene in all 3 varieties (present in 2023) and neral in 2024 in sweet orange.

**TABLE 3:** Mean values (± SD) of the proportion (%) of major LEOs compounds (>1%) from leaves harvested in March 2023 and March 2024 for the 3 citrus: Each value derived from biological triplicates. Statistical significance between young and mature leaves was assessed using Student's T‐test (for normally distributed data) or Mann‐Whitney test (for non‐parametric data). Significant differences (p < 0.05 in light gray, *p* < 0.01 in dark gray) are highlighted in the table.

Compounds	Clementine		Grapefruit		Sweet orange	
	March 2023	March 2024	March 2023	March 2024	March 2023	March 2024
α‐terpinene	0.97 ± 0.39	1.12 ± 0.13	0.11 ± 0.06	1.28 ± 0.08	0.30 ± 0.10	1.29 ± 0.13
γ‐terpinene	2.03 ± 0.89	1.79 ± 0.22	0.43 ± 0.13	2.11 ± 0.14	0.62 ± 0.18	1.46 ± 1.23
limonene	5.85 ± 1.63	3.60 ± 0.32	3.54 ± 0.14	2.61 ± 0.17	3.17 ± 0.47	2.67 ± 0.16
linalool	14.48 ± 3.62	24.67 ± 2.46	8.43 ± 0.14	4.47 ± 1.49	4.09 ± 3.71	5.60 ± 0.36
terpinen‐4‐ol	3.91 ± 2.10	5.35 ± 0.49	1.39 ± 0.17	5.46 ± 0.31	1.85 ± 0.59	5.97 ± 0.50
β‐elemene	0.02 ± 0.05	0.00 ± 0.00	2.94 ± 0.35	0.56 ± 0.06	3.53 ± 0.43	0.61 ± 0.05
*(E)*‐β‐caryophyllene	1.00 ± 0.18	0.13 ± 0.02	1.46 ± 0.20	0.21 ± 0.01	1.16 ± 0.13	0.16 ± 0.01
*(E)*‐β‐farnesene	1.03 ± 0.17	0.03 ± 0.00	0.66 ± 0.49	0.05 ± 0.01	0.72 ± 0.07	0.03 ± 0.00
*(E)*‐phytol	0.24 ± 0.10	0.03 ± 0.04	1.72 ± 0.19	0.26 ± 0.07	1.29 ± 0.23	0.18 ± 0.01
α‐sinensal	1.72 ± 0.15	0.92 ± 0.18	0.03 ± 0.02	0	0.76 ± 0.17	0.23 ± 0.18
β‐sinensal	3.29 ± 0.66	2.13 ± 0.41	1.64 ± 1.38	1.40 ± 0.14	3.04 ± .10	1.27 ± 0.05

The differences between January 2024 and 2025 were minor, and no compounds differed between these two dates in clementine trees (Table 4 and Table S1). Only limonene varied significantly between year in grapefruit (13.80% *vs*. 0.85% in 2024 and 2025 respectively) and in sweet orange (11.20% *vs*. 2.08% in 2024 and 2025 respectively). Other variations among the compounds with a higher proportion than 1%, were very low between the two years.

**TABLE 4:** Mean values (± SD) of the proportion (%) of major LEOs compounds (>1%) from leaves harvested in January 2024 and January 2025 for the 3 citrus varieties: Each value derived from biological triplicates. Statistical significance between young and mature leaves was assessed using Student's T‐test (for normally distributed data) or Mann‐Whitney test (for non‐parametric data). Significant differences (*p* < 0.05 in light gray, *p* < 0.01 in dark gray) are highlighted in the table.

	Clementine		Sweet orange		Grapefruit	
Compounds	Janu‐24	Janu‐25	Janu‐24	Janu‐25	Janu‐24	Janu‐25
(E)‐2‐hexenal	0.68 ± 0.54	0.66 ± 0.18	1.10 ± 0.31	0.49 ± 0.14	0.66 ± 0.27	0.64 ± 0.23
α‐pinene	1.66 ± 0.06	1.43 ± 0.08	1.73 ± 0.11	1.55 ± 0.09	1.82 ± 0.05	1.60 ± 0.19
sabinene	41.38 ± 2.75	38.00 ± 1.08	43.77 ± 4.39	44.29 ± 3.60	43.26 ± 1.48	49.14 ± 0.36
β‐pinene	2.05 ± 0.10	1.87 ± 0.08	2.16 ± 0.18	2.13 ± 0.13	4.13 ± 0.24	4.18 ± 0.65
myrcene	3.00 ± 0.17	2.67 ± 0.08	3.44 ± 0.10	3.01 ± 0.23	3.05 ± 0.17	2.73 ± 0.20
δ‐3‐carene	2.71 ± 0.87	2.02 ± 0.28	3.44 ± 0.88	2.03 ± 0.52	0.04 ± 0.01	0.02 ± 0.00
α‐terpinene	1.56 ± 0.05	1.72 ± 0.35	1.60 ± 0.16	2.15 ± 0.25	1.83 ± 0.11	2.18 ± 0.06
limonene	4.72 ± 1.32	4.35 ± 0.40	11.20 ± 2.33	2.08 ± 0.11	16.8 ± 3.21	1.07 ± 0.91
(*E*)‐β‐ocimene	4.34 ± 0.43	3.39 ± 0.20	5.55 ± 0.53	5.02 ± 0.77	7.46 ± 0.30	7.81 ± 0.64
γ‐terpinene	2.51 ± 0.06	2.76 ± 0.53	2.56 ± 0.24	3.50 ± 0.15	3.03 ± 0.23	3.56 ± 0.04
terpinolene	1.04 ± 0.21	0.94 ± 0.12	1.40 ± 0.15	1.05 ± 0.05	0.67 ± 0.05	0.72 ± 0.06
linalool	24.24 ± 2.07	24.81 ± 3.51	6.20 ± 0.46	8.04 ± 1.16	6.76 ± 1.28	9.48 ± 0.99
citronellal	0.29 ± 0.16	0.65 ± 0.14	1.52 ± 0.33	1.52 ± 0.25	0.31 ± 0.10	0.34 ± 0.11
terpinen‐4‐ol	7.36 ± 0.23	8.22 ± 1.93	7.38 ± 0.73	8.65 ± 0.50	7.75 ± 0.56	8.86 ± 0.99
β‐sinensal	2.18 ± 0.29	1.89 ± 1.30	1.24 ± 0.09	1.98 ± 0.17	1.14 ± 0.10	1.09 ± 0.18
α‐sinensal	0.98 ± 0.18	1.3 ± 0.35	0.37 ± 0.04	0.55 ± 0.06	0	0.06 ± 0.06

Eight compounds varied statistically in the LEO profiles between November 2023 and 2024 but with slight differences (Table 5 and Table S1). The proportion of (E)‐2‐hexenal was higher in 2024 than in 2023 although in 2023 the trees had suffered water stress 2 months earlier. Terpinen‐4‐ol was in higher proportion in 2023 for clementine and grapefruit.

**TABLE 5:** Mean values (± SD) of the proportion (%) of major LEOs compounds (>1%) from leaves harvested in November 2023 and November 2024 for the 3 citrus varieties: Each value derived from biological triplicates. Statistical significance between young and mature leaves was assessed using Student's *t*‐test (for normally distributed data) or Mann‐Whitney test (for non‐parametric data). Significant differences (*p* < 0.05 in light gray, *p* < 0.01 in dark gray) are highlighted in the table.

	Clementine		Sweet orange		Grapefruit	
Compounds	Nov‐23	Nov‐24	Nov‐23	Nov‐24	Nov‐23	Nov‐24
(E)‐2‐hexenal	0.38 ± 0.06	1.03 ± 0.25	0.69 ± 0.25	1.67 ± 0.06	0.73 ± 0.321	2.39 ± 0.48
α‐pinene	1.65 ± 0.10	1.41 ± 0.10	1.98 ± 0.13	1.70 ± 0.12	2.08 ± 0.05	1.72 ± 0.05
sabinene	39.23 ± 1.11	41.42 ± 0.28	49.12 ± 1.18	45.33 ± 2.61	47.05 ± 1.7	44.56 ± 1.67
β‐pinene	1.93 ± 0.04	1.84 ± 0.08	2.36 ± 0.09	2.23 ± 0.09	4.34 ± 0.17	4.33 ± 0.08
myrcene	2.90 ± 0.13	2.62 ± 0.12	3.51 ± 0.01	3.15 ± 0.15	3.13 ± 0.11	2.86 ± 0.12
δ‐3‐carene	3.06 ± 0.68	2.13 ± 0.22	3.25 ± 0.25	2.01 ± 0.33	0.07 ± 0.08	0
α‐terpinene	1.41 ± 0.03	1.42 ± 0.12	1.58 ± 0.00	1.85 ± 0.08	2.02 ± 0.07	1.62 ± 0.16
limonene*	3.00 ± 0.13	4.24 ± 0.27	2.23 ± 0.04	2.35 ± 0.16	2.30 ± 0.20	2.43 ± 0.19
(E)‐β‐ocimene	4.19 ± 0.14	3.37 ± 0.14	5.25 ± 0.08	5.05 ± 0.39	8.67 ± 0.63	8.56 ± 0.69
γ‐terpinene	2.22 ± 0.02	2.33 ± 0.19	2.43 ± 0.01	3.10 ± 0.16	3.21 ± 0.12	2.75 ± 0.21
linalool	23.35 ± 2.47	25.86 ± 2.75	8.99 ± 2.07	8.40 ± 1.28	7.49 ± 1.15	9.12 ± 1.77
citronellal	0.60 ± 0.27	0.80 ± 0.23	1.62 ± 0.23	1.21 ± 0.21	0.63 ± 0.14	0.23 ± 0.05
terpinen‐4‐ol	6.69 ± 0.28	5.60 ± 0.17	7.56 ± 0.47	9.76 ± 1.00	9.13 ± 0.47	6.74 ± 0.65
geranial	0.04 ± 0.01	0.05 ± 0.03	1.24 ± 0.61	0.84 ± 0.22	0	0
β‐sinensal	1.98 ± 0.24	2.67 ± 0.55	1.35 ± 0.27	1.27 ± 0.07	1.19 ± 0.09	1.14 ± 0.97
α‐sinensal	0.82 ± 0.10	1.23 ± 0.65	0.31 ± 0.00	0.37 ± 0.01	0.05 ± 0.07	0.02 ± 0.00

### 2.4 Comparison of LEO Composition Between Adult and Young Leaves

Samples of adult and young leaves were taken 7 days apart. Whatever the variety, LEO composition differed markedly between young growing leaves and adult leaves, especially for 3 major compounds, sabinene, linalool, and terpinen‐4‐ol whatever the citrus varieties (Table 6 and Table S1). The proportion of sabinene was lower in young leaves, while those of linalool and terpinen‐4‐ol were higher. Limonene did not differ between the two leaf maturation stages. The greatest differences between adult and young leaves were observed in sweet orange with eight compounds statistically different between young and mature leaves. A higher proportion of α‐terpinene, γ‐terpinene, and β‐sinensal was detected in young leaf of grapefruit. In sweet orange, the proportion of citronellol and geranial were higher in the LEO of young leaves. Among minor compounds (<1%), τ muurolol and caryophyllene oxide were present only in LEO of adult grapefruit leaves, while 2‐hexanal, bicyclogermacrene, δ‐cadinene, α‐cadinol, and α‐sinensal were specific to young leaves. Among compounds varying in proportion between the two sweet orange leaf stages, citronellal and α‐terpinene were in greater relative proportions in adult leaves, while 11 compounds were in higher quantities in young leaves. In orange, the differences between the two stages for minor compounds were less marked than in grapefruit, but nevertheless (E)‐2‐hexenal and α‐cadinol were again specific to young leaves. Neryl, citronellyl, and geranyl acetates were only present in LEO from adult leaves. Variations between the two stages of leaf maturity were also lower in clementine, but some compounds were specific to each both leaf stages. Geraniol, geranial, α‐humulene and δ‐cadinene were only found in the young leaf, while caryophyllene oxide, citronellyl and geranyl acetates were only present in the adult stage.

**TABLE 6:** Mean percentage (± SD) of the proportion (%) of major LEOs compounds (> 1%) in mature and young leaves harvested at the end of March 2024 on the three citrus varieties: Each value derived from biological triplicates. Statistical significance between young and mature leaves was assessed using Student's *t*‐test (for normally distributed data) or Mann‐Whitney test (for non‐parametric data). Significant differences (*p* < 0.05 in light gray, *p* < 0.01 in dark gray) are highlighted in the table.

	Clementine		Sweet Orange		Grapefruit	
Compounds	Mature	Young	Mature	Young	Mature	Young
sabinene	44.62 ± 4.17	36.21 ± 1.22	55.3 ± 1.85	46.66 ± 2.64	53.56 ± 1.69	42.78 ± 2.20
β‐pinene	2.00 ± 0.21	1.91 ± 0.04	2.41 ± 0.10	2.37 ± 0.31	4.92 ± 0.19	4.88 ± 0.32
myrcene	2.84 ± 0.20	2.84 ± 0.04	3.40 ± 0.06	3.44 ± 0.47	3.03 ± 0.13	3.21 ± 0.04
δ‐3‐carene	2.28 ± 0.46	2.01 ± 0.45	2.42 ± 0.16	1.92 ± 0.34	0.01 ± 0.01	0.02 ± 0.01
α‐terpinene	1.12 ± 0.14	1.97 ± 0.18	1.30 ± 0.13	2.00 ± 0.36	1.29 ± 0.09	2.48 ± 0.39
limonene	3.90 ± 0.32	4.47 ± 0.19	2.67 ± 0.16	2.34 ± 0.31	2.61 ± 0.17	2.41 ± 0.05
(*E*)‐β‐ocimene	0.13 ± 0.01	4.75 ± 0.49	5.25 ± 0.11	6.99 ± 0.88	7.96 ± 0.82	9.14 ± 0.40
γ‐terpinene	3.83 ± 0.10	3.17 ± 0.30	2.13 ± 0.09	3.23 ± 0.60	2.11 ± 0.14	4.06 ± 0.62
*trans*‐sabinene hydrate	1.79 ± 0.22	0.69 ± 0.04	1.16 ± 0.98	0.72 ± 0.17	0.44 ± 0.02	0.55 ± 0.04
p‐cymenene	1.07 ± 0.19	0	0	0	0.03 ± 0.00	0.06 ± 0.02
terpinolene	0.75 ± 0.06	1.05 ± 0.04	1.78 ± 0.28	1.41 ± 0.46	0.49 ± 0.03	1.02 ± 0.10
linalool	24.68 ± 2.46	29.65 ± 1.55	5.60 ± 0.37	11.63 ± 1.71	4.48 ± 1.49	14.41 ± 0.95
citronellal	0.54 ± 0.49	0.13 ± 0.06	1.79 ± 0.28	0.26 ± 0.05	0.28 ± 0.10	0.04 ± 0.01
terpinen‐4‐ol	5.35 ± 0.50	0	5.98 ± 0.50	9.06 ± 1.45	5.47 ± 0.32	11.13 ± 1.92
α‐terpineol	0.64 ± 0.18	1.74 ± 0.12	0.28 ± 0.01	0.67 ± 1.08	0.21 ± 0.01	0.01 ± 0.01
citronellol	0.05 ± 0.01	0.10 ± 0.05	0.69 ± 0.13	1.41 ± 0.46	0	0.14 ± 0.06
geranial	0	0.13 ± 0.06	0.87 ± 0.17	1.93 ± 0.51	0.01 ± 0.02	0.18 ± 0.07
β‐sinensal	2.13 ± 0.41	2.01 ± 0.58	1.27 ± 0.05	1.82 ± 0.60	1.4 ± 0.15	2.23 ± 0.16
α‐sinensal	0.93 ± 0.18	1.60 ± 0.42	0.23 ± 0.18	0.90 ± 0.25	0	0.23 ± 0.16

## 3 Discussion

Our study is based on a plot where trees of all citrus varieties are cultivated in exactly the same way each year. Outside the water deficient period and with the exception of sabinene and limonene, the fluctuation of the various compounds was very low or even often non‐existent, from 1 year to the next, whatever the variety. This observation is in agreement with a previous study where the compositions of citrus essential oils remained largely unchanged over a 20‐year period [2]. However, change in the proportion of certain compounds were detected only once, such as γ‐elemene in March 2023 and octanal in September 2022 in grapefruit and sweet orange. Climatic factors (temperature and hygrometry) fluctuate much more during the year than between years in our experimental period. The monthly variation of essential oil was previously observed in sour orange (*C. aurantium*) grown in Tunisia [4]. Environmental parameters such as temperature, relative humidity, total duration exposure to sun and wind patterns are supposed to have a direct influence especially in species that have histological structures for the storage of essential oil on the leaf surface [12, 13, 14].

We decided to conduct our observations on three citrus varieties widely cultivated in the Mediterranean basin, as well as around the world in the case of oranges. It can also be stated that the results described can be generalized to all cultivars of these three varieties, as the genetic diversity among cultivars is very low. The cultivars were selected from bud mutations that alter very few traits [3, 15]. Human selection has retained only a few innovative phenotypes such as the pulp bloody coloration of few orange cultivars [16]. This cultivar selection was not based on aromatic characteristics or LEO composition, as demonstrated in sweet orange trees [17]. The range of variation in orange and grapefruit The LEO pattern of sweet orange and grapefruit were similar to that observed in the literature [18], notably for sabinene, linalool and terpineol‐4‐ol. Only limonene variation is significantly lower in literature than that observed in the current experiment.

Although the trees are native to tropical or subtropical regions, we cannot confirm that they were under physiological stress during the winter months. However, the stop of irrigation in August 2023 caused the leaves to curl (epinasty) after three weeks, a well‐known sign of water stress in trees. To confirm this state of stress in the trees, physiological measurements (photosynthetic activity, stomatal conductance, photosystem II status, etc.) would have been necessary. However, although temperatures are practically always positive. we suppose that the climatic conditions of the winter period in Corsica represent a moderate stress for the 3 citrus varieties. This assertion was supported by a study developed in the same experimental orchard (in Corsica) where the physiological activities of citrus species between winter and summer periods that showed a decline in photosynthetic parameters, accumulation of oxidative compounds (Reactive Oxygen Species) and increase in antioxidant performance in cold period [19]. The authors concluded that all trees were subjected to a chilling oxidative stress even if the temperatures were always positive but lower to 10°C.

Sabinene and limonene are the two main compounds in citrus LEO that respond to environmental variations and especially to water stress during our current experience. We can therefore assume that there is a cause‐and‐effect relationship between these two variables. The literature contains numerous examples describing the correlation between water stress and changes in the proportions of monoterpenes and sesquiterpenes [6, 20, 21, 22, 23, 24, 25, 26, 27, 28]. Drought stress induces oxidative stress at the cellular and intercellular level with a production of reactive oxygen species (ROS) [29]. Since secondary metabolites possess strong antioxidative properties, it may be related to a mechanism to counteract the deleterious effects of ROS [30]. Stimulation of EO production under water stress produces high terpene levels in plants leaves because of a lesser allocation of carbon to growth, suggesting a trade‐off between growth and defense [31]. Water stress applied to lemon trees was found to correlate with improved stress tolerance in the trees characterized by significantly higher relative proportions of α‐pinene, sabinene, myrcene, limonene, and ocimene [9]. The authors suggested a higher protection of cell membrane integrity could be associated with the higher amounts of monoterpenes

The proportion of (E)‐2‐hexenal in our experiment increased in winter (November to January) and during water deficit in September and October 2023 and it was correlated to period of stress. This corroborates observations in the literature, which mention that (E)‐2‐hexenal and other oxylipins are signaling molecules that regulate defense in many plants in biotic and abiotic stresses such as water stress [9, 32, 33]. Although constitutively produced, secondary metabolite synthesis is increased in situations where plants are subjected to attack [34]. Volatile aliphatic compounds as well as aldehydes, alcohols and their esters are emitted or increased by tissues exposed to biotic and abiotic stress, to limit the consequences of stress or to communicate from plant to plant [35, 36].

The stage of development of plant organs has a significant impact on the composition of essential oils. This is demonstrated in our study by the composition of essential oils in young leaves and mature leaves. The monoterpenes (sabinene, terpinen‐4‐ol, linalool) were the most variable aromatic compounds according to leaf age. The proportion of sesquiterpenes, was always higher in young leaves, of grapefruit (4.00% *vs*. 1.95%), sweet orange (3.90% *vs*. 2.43%) and clementine (4.11% *vs*. 3.39%). This is agreement with the result of a study conducted on sweet orange volatile compounds from juvenile and mature intact leaves [37]. On the other hand, the stage of fruit development has often been described as a major factor of variation in Peel essential oil composition. The early stages of fruit development were characterized by a significant increase of oxygenated monoterpenes [3]. The majority of compounds varying according to leaf age have characteristic odors [38]. We can therefore assume that the aroma profile of LEO must differ according to leaf age.

## 4 Conclusions

The dwindling water resources and the associated water stress, are major concerns for crops exposed to climate change, particularly in the Mediterranean climate, where summers are becoming drier and hotter. Adapting plants to these climatic changes is at the heart of our research. The impact of climate change on the composition of essential oils is a widespread question among users of aromatic plants. In citrus, the environment, which includes both climate and cultivation techniques, has a real impact on essential oil composition and aroma pattern.

Citrus essential oils are often presented as compounds involved in thermal regulation and, above all, as chemical messengers inducing physiological reactions in trees under stress. Our study showed that the proportion of some LEO monoterpenes (sabinene and limonene) and one linear aldehyde ((E)‐2‐hexenal) fluctuated according to the seasons, but more intensively during water stress. The intensity of this tree reaction varied according to species (orange and grapefruit more reactive to water stress than clementine and vice versa for winter weather). Further studies could be conducted to test the effectiveness of these compounds in helping trees adapt to climate stress under controlled conditions, and thus assess whether they could serve as selection markers for plant breeding. The results of our study have another implication: they demonstrate the instability of the composition of essential oils derived from leaves, which is subject to seasonal climatic variations and, consequently, to changing aromatic properties. Our study provides guidance to growers on the period and climatic conditions to be taken into account to control and stabilize the LEO quality.

## 5 Experimental Section

### 5.1 Citrus Trees

Clonal propagated trees were maintained in the INRAE‐Cirad citrus germplasm (BRC ISO 9001), located at San Giuliano, Corsica, France (latitude 42°170 N, longitude 9°320 E). Observations were carried out on 3 varieties: Tomatera clementine (*Citrus x aurantium* var. *clementina*; SRA 535), Star Ruby grapefruit (*C. x aurantium* var. *paradisii*; SRA 293) and Cara Cara Navel sweet orange (*C. aurantium* var. *sinensis*; SRA 666). These three varieties are economically significant citrus fruits because they are grown in many countries around the world, particularly in the Mediterranean area. All trees are grafted onto the same rootstock, Carrizo citrange (*Poncirus trifoliata* X *C. sinensis* or *C. trifoliata* X *C. x aurantium* var. *sinensis*), and grown in a same orchard under the same conditions [39]. The plantation was laid out in the same way as commercial orchards, with a spacing of 4 meters between trees and 6 meters between rows. The trees are pruned annually to maintain a rounded canopy no more than 2.5 meters high. The ground is covered with grass and mowed once a month during the growing season. The trees are 22 years old and have been bearing fruit for over 17 years. The experiment was conducted in September 2022, from September 2023 to March 2024, in September 2024 and in January 2025. In summer 2023, irrigation stopped at mid‐August to measure the effects of summer drought on LEO composition and the other years water supply was maintained until October after the first autumn rainfalls. Between the 12^th^ and 16^th^ of each month, 400 g of adult leaves per tree and on 3 trees for each variety, were taken from the east‐facing side. On 30^th^ March 2024, 400 g of young immature leaves from the first flush of spring were harvested under the same conditions as before. The leaves were frozen at −20°C before extraction of the essential oil (LEO).

### 5.2 Climatic Data

**FIGURE 3:**
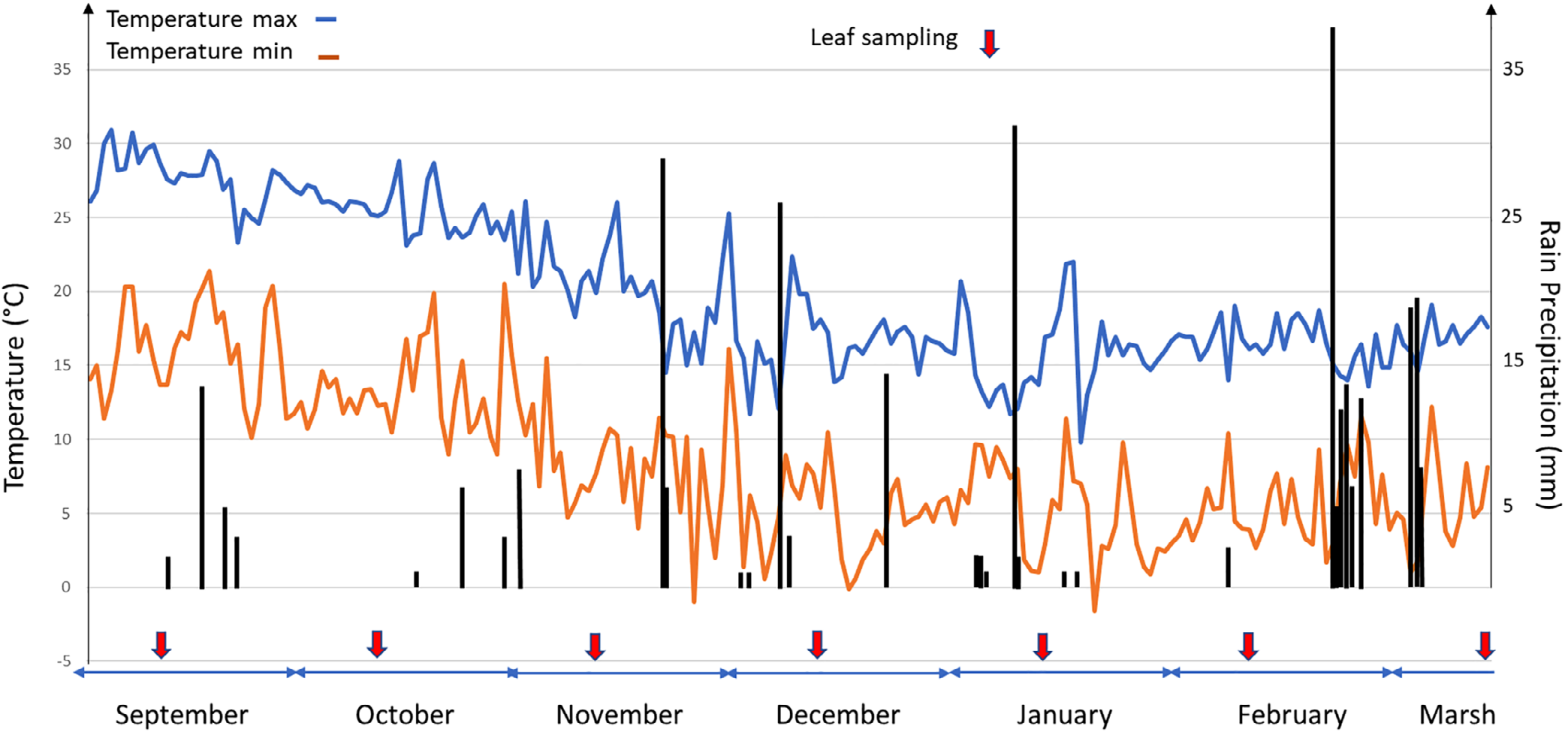
Daily values of minimum (red) and maximum (blue) temperatures in °C and rainfall in mm, over the period from September 1, 2023 to March 18, 2024. Red arrows indicate leaf sampling dates.

The INRAE weather station at San Giuliano is located close to the experimental orchard and is part of an INRAE network of agroclimatic stations. The stations measure agroclimatic variables for field research infrastructures (SOERE, crop system trials, phenotyping platform, etc.). Minimum, maximum, and average temperatures (°C), rainfall (mm), photosynthetically active radiation (PAR in µmol.m^−2^.s‐1) and Potential evapotranspiration (PET in mm) were daily recorded. The date of leaf sampling, the daily rain precipitation and the minimum and maximum temperatures from September 2023 to March 2024 are presented in Figure 3.

### 5.3 Leaf Essential Oil (LEO) Extraction

All sampling was performed in biological replicates, consistently using the same trees throughout the experiment. For each replicate, 120 g of fresh leaves were subjected to water distillation for 2 h 30 using a Clevenger‐type apparatus in 2 L flask with 1 L of distilled water. To avoid any damage, the LEO samples were stored at +5°C in amber vials until chromatographic analysis.

The method, protocol, and apparatus for essential oil analysis are identical to the team's previous publication [3].

### 5.4 Gas Chromatography (GC) Analysis

Each sample was analyzed by dual column gas chromatography and gas chromatography combined with mass spectrometry (GC‐MS) in order to determine the chemical composition. GC analyses were performed on a Clarus 500 FID gas chromatograph (PerkinElmer, Courtaboeuf, France) equipped with two fused silica gel capillary columns (length 50 m, internal diameter 0.22 mm and film thickness 0.25 µm), BP‐1 (polydimethylsiloxane) and BP‐20 (polyethylene glycol). The oven temperature was programmed to increase from 60 to 220°C at 2°C/min and then held in an isothermal state at 220°C for 20 min, injector temperature: 250°C; detector temperature: 250°C; carrier gas: hydrogen (1.0 mL/min); and split: 1/60. The relative proportions of the oil constituents were expressed as percentages obtained by peak area normalization without using correcting factors. Retention indices (RIs) were determined relative to the retention times of a series of n‐alkanes with linear interpolation (‘Target Compounds’ software of PerkinElmer, Courtaboeuf, France). The essential oil (EO) samples (30 mg) were diluted in 0.5 mL of chloroform_._

### 5.5 Mass Spectrometry

EOs were analyzed with a PerkinElmer TurboMass detector (quadrupole, Perkin Elmer, Courtaboeuf, France), coupled directly to a PerkinElmer Autosystem XL (PerkinElmer), equipped with a fused silica gel capillary column (length, 50 m; internal diameter, 0.22 mm; film thickness, 0.25 µm), and BP‐1 (polydimethylsiloxane). Helium was used as carrier gas at 0.8 mL/min, 1/75 split injection and 0.5 µL was injected. The injector temperature was 250°C. The oven temperature was programmed to increase from 60 to 220°C at 2°C/min and then held in an isothermal state for 20 min. The ion source temperature and energy ionization were set at 250°C and 70 eV, respectively. Electron ionization mass spectra were acquired over a 40–400 Da mass range. Oil samples were diluted in chloroform with 30 mg of essential oil in 0.5 mL of chloroform.

### 5.6 NMR Analysis

^13^C NMR analyses were performed on an AVANCE 400 Fourier transform spectrometer (Bruker, Wissembourg, France) operating at 100.623 MHz for ^13^C, equipped with a 5 mm probe, in CDCl_3_, with tetramethylsilane (TMS) used as internal reference. ^13^C NMR spectra were recorded with the following parameters: pulse width (PW): 4 µs (flip angle 45°); acquisition time: 2.73 s for 128 K data table with a spectral width (SW) of 220.000 Hz (220 ppm); CPD mode decoupling; and digital resolution 0.183 Hz/pt. The number of accumulated scans ranged from 2000–3000 per sample (≈ 30 mg of oil in 0.5 mL of CDCl_3_). Exponential line broadening multiplication (1.0 Hz) of the free induction decay was applied before Fourier transformation.

### 5.7 Identification of Individual Components

The components were identified via three methods. The first one was a comparison of their GC retention indices (RIs) on polar and apolar columns, determined relative to the retention times of a series of *n*‐alkanes with linear interpolation (‘Target Compounds’ software of PerkinElmer), with those of authentic compounds. The second one was based on computer matching against commercial mass spectral libraries and by comparison of spectra with literature data. The last method, used for few samples, was based on a comparison of the signals in the ^13^C NMR spectra of EOs with those of reference spectra compiled in the laboratory spectral library. In the investigated samples, NMR identified individual components at contents as low as 0.5%.

### 5.8 Statistical Analysis

Statistical analyses were performed in R (v 4.4.3) using an adaptive approach to compare percentages between two groups (dates or types): Each value derived from biological triplicates. For each compound and species, normality was assessed using the Shapiro‐Wilk test (*p* > 0.05), and homogeneity of variances was tested with Levene's test (*p* > 0.05). Depending on the results, either a student's t‐test (pooled) or a Wilcoxon rank‐sum test (Mann‐Whitney) was applied to evaluate significant differences.

## Author Contributions

F.L conceived, designed the experiment, harvested the leaves, analyzed the data and wrote the manuscript. M.R. realized the acquisition of chemical data. M.P provided the technical support for the chromatography and mass spectrum analysis. F.T. analyzed the data and wrote the manuscript. All authors have read and agreed to the published version of the manuscript.

## Funding

This work has received research funding from the French government managed by the National Research Agency under the France 2030 program, with reference number ANR‐22‐EXES‐0016.

## Conflicts of Interest

The authors declare no conflict of interest.

## Supporting information




**Supporting file**: cbdv71216‐sup‐0001‐SuppMat.xlsx

## Data Availability

The data supporting the findings of this study are available from the corresponding author upon request.
